# Anti-Fibrosis Effect of *Panax ginseng* and *Inula japonica* Formula in Human Pulmonary Fibroblasts

**DOI:** 10.3390/nu16020319

**Published:** 2024-01-22

**Authors:** YeonGyun Jung, Nam-Hui Yim, Sang Myung Lee, Won-Kyung Cho, Min Ho Cha, Jin Yeul Ma

**Affiliations:** 1 Burn Institute, Department of Rehabilitation Medicine, Hangang Sacred Heart Hospital, Hallym University College of Medicine, Seoul 07247, Republic of Korea; j1076@naver.com; 2 Korean Medicine (KM) Application Center, Korea Institute of Oriental Medicine, Daegu 41062, Republic of Korea; wkcho@kiom.re.kr (W.-K.C.); mhchamin@kiom.re.kr (M.H.C.); 3 Division of Food and Pharmaceutical Technology, College of Health and Safety Science, Mokwon University, Daejeon 35349, Republic of Korea; smlee@mokwon.ac.kr

**Keywords:** pulmonary fibrosis, *Inula japonica*, *Panax ginseng*, transforming growth factor-β1, fibroblast-to-myofibroblast transition

## Abstract

*Panax ginseng* Meyer and *Inula japonica* Thunb. are well established in traditional medicine and are known for their therapeutic properties in managing a range of ailments such as diabetes, asthma, and cancer. Although *P. ginseng* and *I. japonica* can alleviate pulmonary fibrosis (PF), the anti-fibrosis effect on PF by the combination of two herbal medicines remains unexplored. Therefore, this study explores this combined effect. In conditions that were not cytotoxic, MRC-5 cells underwent treatment using the formula combining *P. ginseng* and *I. japonica* (ISE081), followed by stimulation with transforming growth factor (TGF)-β1, to explore the fibroblast-to-myofibroblast transition (FMT). After harvesting the cells, mRNA levels and protein expressions associated with inflammation and FMT-related markers were determined to evaluate the antiinflammation activities and antifibrosis effect of ISE081. Additionally, the anti-migratory effects of ISE081 were validated through a wound-healing assay. ISE081 remarkably reduced the mRNA levels of interleukin (IL)-6, IL-8, α-smooth muscle actin (SMA), and TGF-β1 in MRC-5 cells and suppressed the α-SMA and fibronectin expressions, respectively. Furthermore, ISE081 inhibited Smad2/3 phosphorylation and wound migration of MRC-5 cells. Under the same conditions, comparing those of ISE081, *P. ginseng* did not affect the expression of α-SMA, fibronectin, and Smad2/3 phosphorylation, whereas *I. japonica* significantly inhibited them but with cytotoxicity. The results indicate that the synergistic application of *P. ginseng* and *I. japonica* enhances the anti-fibrotic properties in pulmonary fibroblasts and concurrently diminishes toxicity. Therefore, ISE081 has the potential as a prevention and treatment herbal medicine for PF.

## 1. Introduction

Pulmonary fibrosis (PF), an enduring and progressively worsening interstitial lung disease with fibrotic features, is marked by the proliferation of lung fibroblasts and the accumulation of the extracellular matrix (ECM) [1,2]. The pathogenesis of idiopathic pulmonary fibrosis (IPF) is still not fully understood. However, it is believed that IPF may result from an abnormal response of fibroblasts to repeated damage of lung epithelial cells and subsequent inefficient repair processes [3]. Significantly, the initiation and persistence of the fibrotic process could be prominently influenced by inflammatory cytokines. The cytokine TGF-β can act as an important growth factor that activates fibroblasts and thus induces phenotypic metastasis of myofibroblasts [4]. For example, TGF-β1 activates lung fibroblasts and plays a role in ECM proliferation, migration, trans-differentiation, synthesis, and deposition. While the activation of Smad2/3 by TGF-β1 plays a pivotal role in initiating the epithelial–mesenchymal transition (EMT) [5,6], this mediator is also capable of triggering non-Smad signaling pathways. These include the mitogen-activated protein kinase (MAPK) pathways, encompassing p38, c-Jun *N*-terminal kinase (JNK), extracellular signal-regulated kinase (ERK), and the phosphatidylinositol-3 kinase (PI3K)/Akt pathway. Because effective treatment methods for PF are currently unavailable, new pulmonary fibrosis treatment studies are required [7]. Herbal medicine’s healthcare efficacy has received much attention in recent years. Traditional medicine (TM), a blend of several herbs, is believed to work synergistically and reduce side effects and toxicity through herb–herb interactions [8]. Notably, single herbs and TM formulas have potential benefits for IPF treatment [9]. *Panax ginseng* Meyer and *Inula japonica* Thunb. are herbal medicines that have long been used to treat various diseases. *I. japonica* is traditionally utilized in the management of conditions such as coughs, gastrointestinal disturbances, and bronchitis [10]. The *I. japonica* extract has recently been shown to have beneficial pharmacological effects, such as antidiabetic, antihyperlipidemic, antiallergic, and antiasthmatic properties [11,12,13,14]. Furthermore, it demonstrates inhibitory effects on pulmonary fibrosis in both in vivo and in vitro studies [15,16]. Furthermore, *P. ginseng* has been traditionally utilized for its efficacy in alleviating fatigue and weakness [17], along with treating a variety of conditions including cancer, Alzheimer’s disease, cognitive impairments, and respiratory illnesses [18]. The combined therapeutic potential of *P. ginseng* and *I. japonica*, however, remains largely unexplored. In the present study, we determined the effect of ISE081, the formula combining *P. ginseng* and *I. japonica*, in treating pulmonary fibrosis using a TGF-β1-stimulated MRC-5 human lung fibroblasts by comparing the anti-fibrosis effects of each herbal medicine.

## 2. Materials and Methods

### 2.1. Chemicals and Reagents

For cell cultivating, minimal essential medium (MEM) with Earle’s balanced salt solution (EBSS) containing 2.0 mM L-glutamine, fetal bovine serum (FBS), and antibiotics were purchased from Hyclone (Logan, UT, USA). Human transforming growth factor-β1 human (TGF-β1), bovine serum albumin (BSA), and dimethyl sulfoxide (DMSO) were obtained from Sigma-Aldrich (St. Louis, MO, USA). Cell culture dishes and well plates were purchased from Sarstedt (Nümbrecht, Germany). For cell viability measurement, the cell-counting kit (CCK) was obtained from Dojindo (Kumamoto, Japan). The alpha-smooth muscle actin (α-SMA) antibody and fibronectin antibody were obtained from Abcam (Cambridge, UK) and Santa Cruz Biotechnology (Santa Cruz, CA, USA), respectively. Alpha-Tubulin, E-cadherin, p38 MAPK, phosphor-p38 MAPK (Thr180/Tyr182), p44/42 MAPK (ERK1/2), phospho-p44/42 MAPK (Erk1/2) (Thr202/Tyr204), SAPK/JNK, phosphor-SAPK/JNK (Thr183/Tyr185), Smad2/3, and Phospho-Smad2 (Ser465/467)/Smad3 (Ser423/425) antibodies were purchased from Cell Signaling Technology (Danvers, MA, USA). The secondary antibodies, including anti-rabbit and anti-mouse, Alexa Fluor 488-conjugated anti-rabbit secondary antibody, and Trizol reagent, were purchased from Invitrogen (Carlsbad, CA, USA). Oligonucleotide primers for real-time reverse transcription-qPCR, DNA synthesis kits, and qPCR Master Mix (AccuPower^®^ 2x GreenStar) were purchased from Bioneer (Daejeon, Republic of Korea).

### 2.2. Preparation of ISE081

*P. ginseng* and *I. japonica* were acquired from Humanherb (Daegu, Republic of Korea) and were preserved at the KM Application Center’s herbal bank in KIOM, under the herbal material number 081. To prepare ISE081, two herbal formulas mixed in a ratio of 1.5 to 1 was extracted by 70% ethanol of 6–8 times herb weight at 37 °C for 24 h with mild shaking. Each herbal material, *P. ginseng,* and *I. japonica* was also extracted under the same condition as ISE081. After being filtered through standard 150 μm pore-size test sieves from Retsch (Haan, Germany), the extracts were then concentrated into a dry form using a lyophilizer. Subsequently, these dried extracts were reconstituted in a solution of 50% DMSO and water before being applied to the cells.

### 2.3. HPLC Analysis of ISE081

Standardization of ISE081 was performed via high-performance liquid chromatography (HPLC) fingerprinting using the maker compounds isolated from *P. ginseng* (ginsenoside Rg1 and ginsenoside Re) and *I. japonica* (britannilactone, 1-*O*-Acetylbritannilactone, and 2-α-Hydroxyeudesma-4,11(13)-dien-12,8β-olide), respectively. Standard solutions and analyzed extracts were prepared by dissolving each marker component in 70% ethanol at 10 mg/mL. For sample analysis, the Agilent 1200 Series gradient HPLC system (Agilent Technologies, Santa Clara, CA, USA) equipped with Discovery C18 column (4.6 × 250 mm, 5 μm, Supelco, Bellefonte, PA, USA) was used and the column was maintained at 30 °C during being analyzed. Detective UV wavelengths were 203 nm and 225 nm based on the UV spectrum of each component in ISE081. The elution conditions consisting of water (A) and acetonitrile (B) at a flow rate of 1.0 mL/min were as follows: 15–20% B, 0–20 min; 20–25% B, 20–30 min; 25–30% B, 30–40 min; 30–45% B, 40–50 min. A volume of 10 μL was used for each sample injection. The data acquisition and analysis were conducted using Agilent ChemStation LC B.04.03 Software.

### 2.4. Cell Culture and Treatment

MRC-5 cells were cultured in minimal essential medium (MEM) supplemented with Earle’s balanced salt solution (EBSS), 2.0 mM L-glutamine, 1% antibiotics, and 10% fetal bovine serum (FBS), and they were maintained at 37 °C in a 5% CO_2_ humidified atmosphere. Upon reaching 80–90% confluence, the cells were subjected to starvation using media containing 1% FBS for a duration of 1 h. Prior to further experimentation, cells underwent a pre-treatment phase where they were exposed either to ISE081 (at concentrations of 25, 50, or 100 mg/mL), *P. ginseng* (60 or 100 mg/mL), or *I. japonica* (40 or 100 mg/mL) for a period of 2 h. The cells were incubated with or without TGF-β1 (5 ng/mL), and each sample was harvested at the indicated intervals. The same volume of solvent was added to the sample instead of the drug or TGF-β1 for each sample for the negative control.

### 2.5. Cell Viability

The cytotoxic effects of IS were assessed using a CCK assay. For this, MRC-5 cells were plated at a concentration of 2 × 10^5^ cells per well in 96-well plates and allowed to settle for 24 h. They were then treated with varying concentrations of IS, *P. ginseng*, *I. japonica*, or TGF-β1. Following a 24 h incubation period, CCK solution was added to each well and the plates were incubated at 37 °C for an additional hour. Cell viability was then measured based on the optical density at 450 nm, using an ELISA plate reader.

### 2.6. Total RNA Extraction and RT-qPCR

Using Trizol reagent as per the guidelines provided by the manufacturer, total RNA was extracted from MRC-5 cells. Subsequently, the quality and quantity of the extracted RNA were assessed using a Nanodrop 2000 instrument (Thermo Scientific, Wilmington, DE, USA). A total RNA (1 μg) was reverse transcribed into cDNA by AccuPower^®^ RT PreMix (Bioneer, Daejeon, Republic of Korea). The primers used for PCR were as follows (from 5 to 3): α- SMA, CTATGCCTCTGGACGCACAACT (forward) and CAGATCCAGACGCATGATGGCA (reverse); COL1A1, GATTCCCTGGACCTAAAGGTGC (forward) and AGCCTCTCCATCTTTGCCAGCA (reverse); COL3A1, TGGTCTGCAAGGAATGCCTGGA (forward) and TCTTTCCCTGGGACACCATCAG (reverse); IL-6, AGACAGCCACTCACCTCTTCAG (forward) and TTCTGCCAGTGCCTCTTTGCTG (reverse); IL-8, GAGAGTGATTGAGAGTGGACCAC (forward) and CACAACCCTCTGCACCCAGTTT (reverse); TGF-β1, TACCTGAACCCGTGTTGCTCTC (forward) and GTTGCTGAGGTATCGCCAGGAA (reverse); GAPDH, GTCTCCTCTGACTTCAACAGCG (forward) and ACCACCCTGTTGCTGTAGCCAA (reverse). RT-qPCR was conducted with a CFX96 System (BIO-RAD, Contra Costa, CA, USA) using the AccuPower^®^ 2× Greenstar qPCR master mix (Bioneer, Daejeon, Republic of Korea). The RNA expression was determined using the 2^−ΔΔCt^ method and normalized based on GAPDH expression. All experiments were performed in triplicate.

### 2.7. Immunoblot Analysis

MRC-5 cells were disrupted in radioimmunoprecipitation assay (RIPA) lysis buffer containing protease and phosphatase inhibitors (Millipore, Bedford, MA, USA) and incubated on ice for 30 min. The lysates were then centrifuged at 14,000× *g* for 20 min at 4 °C. Protein concentrations were determined using the Bradford assay. Samples containing 50 μg of protein were separated by 8 or 12% SDS-PAGE and then transferred onto PVDF membranes. For blocking, 3% BSA in TBST was used for 1 h at room temperature, followed by overnight incubation at 4 °C with specific primary antibodies. The membranes were then washed in TBS with 0.1% Tween 20 and subsequently incubated with HRP-conjugated secondary antibodies for 1 h at room temperature. Chemiluminescence was used to detect specific proteins, and protein expression levels were semi-quantitatively analyzed using ImageJ software version 1.53k (https://imagej.nih.gov/ij/, accessed on 6 July 2021).

### 2.8. Immunofluorescence Staining

After 24 h of treating MRC-5 cells with TGF-β1 along with ISE081 concentrations of 25, 50, and 100 μg/mL, the cells underwent a washing step with PBS. Subsequently, they were fixed using 3.7% formaldehyde solution and permeabilized with 0.1% Triton X-100 in PBS. Blocking was conducted using 3% bovine serum albumin (BSA) in PBST. Overnight incubation with α-SMA antibodies was followed by staining with Alexa Fluor 594-labeled secondary antibodies for one hour. Nuclei of the cells were colored blue using 4,6-diamidino-2-phenylindole (DAPI) while α-SMA expression was visualized in red through immunofluorescence microscopy.

### 2.9. Wound-Healing Assay

In the experiment, MRC-5 cells were grown to full confluency in 12-well plates. A cell scraper was then used to create a scratch across each well’s cell layer. Following this, the monolayer was rinsed thrice with PBS to eliminate detached cells and debris. Each well was treated with 1 mL of MEM containing 1% FBS and EBSS, supplemented with 2.0 mM L-glutamine, and varying concentrations of ISE081 (25, 50, or 100 μg/mL) along with TGF-β1. The cells were then incubated at 37 °C in an atmosphere of 5% CO_2_ for 24 h. Microscopic imaging was performed to record the scratch area both prior to and following incubation periods of 16 and 24 h, ensuring the same field of view for each time point. Scratch width comparisons were conducted using an enhanced version of Image J plugin software version 1.53k [19]. Migration levels were quantified by assessing wound healing at 24 h post-treatment. The percentage of wound closure for each treatment condition was calculated and normalized against the control group’s wound closure percentage. This procedure was replicated three times for consistency. Before and after the 24 h incubation period, additional images were taken at identical positions to measure and compare scratch closure percentages. This set of experiments was also conducted in triplicate.

### 2.10. Statistical Analysis

All statistical analyses in the study were carried out using Prism 8.0 software (GraphPad, San Diego, CA, USA). To evaluate multiple comparisons, a one-way ANOVA was initially performed, followed by Tukey’s HSD post hoc test for detailed analysis. The threshold for statistical significance against the specified control group was set at a *p*-value of less than 0.05.

## 3. Results

### 3.1. Effect of ISE081 on Cell Viability in MRC-5 Cells

**Figure 1:**
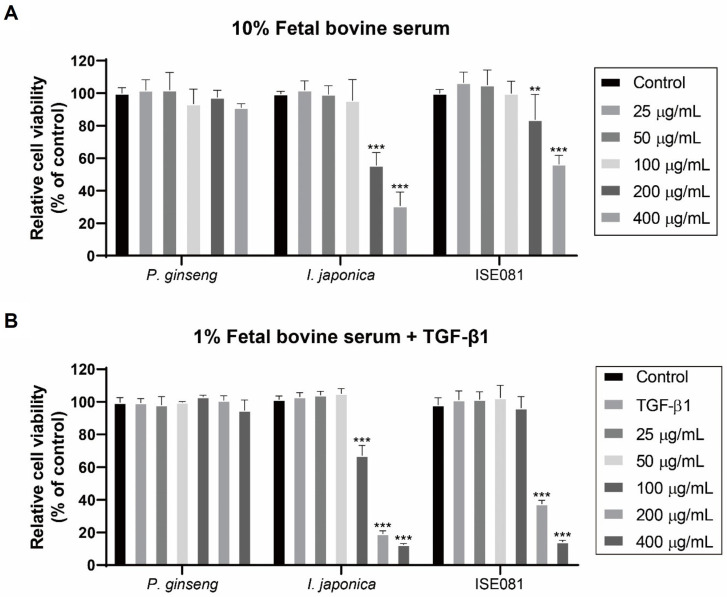
Cell viability after treatment with ISE081 and its components (*P. ginseng* and *I. japonica*). CCK-9 assay showing the relative cell viability (%) in MEM media containing 10% FBS (**A**) or 1% FBS and 5 ng/mL TGF-β1 (**B**) after 24 h. Results are normalized to the control (cells without treatment). Data are presented as mean SD, *n* = 5 per group. ** *p* < 0.01, *** *p* < 0.001 vs. control group.

Cell viability was assessed using a CCK-8 assay following a 24 h incubation with ISE081 and its components (*P. ginseng* and *I. japonica*) at varying concentrations in MEM media containing 10% FBS. At all concentrations, *P. ginseng* did not differ from the control in terms of cell viability. However, for *I. japonica* and ISE081, cell viability decreased significantly at concentrations of 200 μg/mL or more (Figure 1A). In case of measuring the cell viability after treating the MRC-5 cells with the extracts in MEM media supplemented with 1% FBS and TGF-β1 (5 ng/mL) for 24 h, *P. ginseng* treatment still showed no significant difference. Cell viability was notably reduced when treated with ISE081 at concentrations of 200 μg/mL or higher, similar to observations in media containing 10% FBS. However, treatment with *I. japonica* demonstrated a significant reduction in cell viability at a lower concentration of 100 μg/mL (Figure 1B). As a result, an antifibrosis experiment was performed by applying the concentration of ISE081 at 100 μg/mL not showing cytotoxicity.

### 3.2. ISE081 Attenuates TGF-β1 Induced Inflammation and α-SMA in MRC-5 Cells

**Figure 2:**
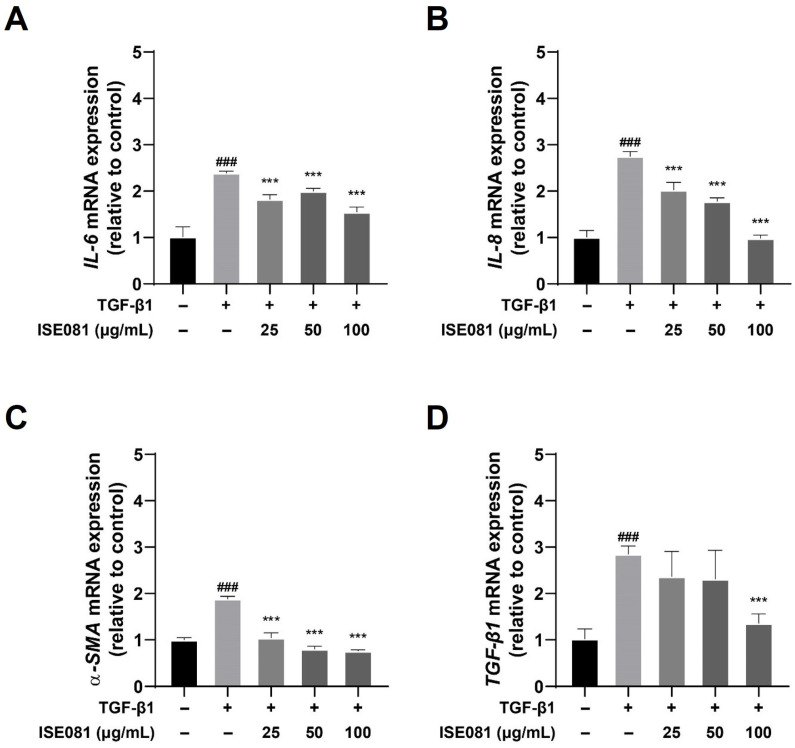
Influence of ISE081 on the expression of IL-6, IL-8, α-SMA, and TGF-β1 in MRC-5 cells activated by TGF-β1. MRC-5 cells underwent pretreatment with various concentrations of ISE081 before incubation with 5 ng/mL TGF-β1 for a period of 6 h. The mRNA levels of IL-6 (**A**), IL-8 (**B**), α-SMA (**C**), and TGF-β1 (**D**) were quantified through RT-PCR. Expression levels were normalized to GAPDH using the 2^−ΔΔCt^ method. The presented data represent the mean ± SD for six replicates per group. Statistical significance is denoted as ### *p* < 0.001 compared to the control group, and *** *p* < 0.001 compared to the group treated with TGF-β1 alone.

In this study, we examined the effects of ISE081 on pulmonary fibrosis, focusing particularly on the expression of proinflammatory cytokines and α-SMA, which were quantified using RT-qPCR. As depicted in Figure 2, treatment with TGF-β1 resulted in an upregulation of IL-6, IL-8, α-SMA, and TGF-β1 mRNA levels compared to the control group. Intriguingly, the co-treatment with ISE081 for a duration of 6 h seemed to mitigate this upregulation. A significant reduction in IL-6 mRNA expression was observed in cells treated with ISE081 at concentrations of 25, 50, and 100 μg/mL, relative to cells treated solely with TGF-β1, as shown in Figure 2A. Furthermore, the levels of IL-8 and α-SMA also demonstrated substantial decreases across all ISE081 concentrations, suggesting a dose–response relationship (see Figure 2B,C). Most notably, the highest concentration of ISE081 (100 μg/mL) resulted in a significant diminution of TGF-β1 mRNA expression when compared to the TGF-β1 only treatment group, as evidenced in Figure 2D.

### 3.3. ISE081 Suppresses TGF-β1 Induced EMT in MRC-5 Cells

**Figure 3:**
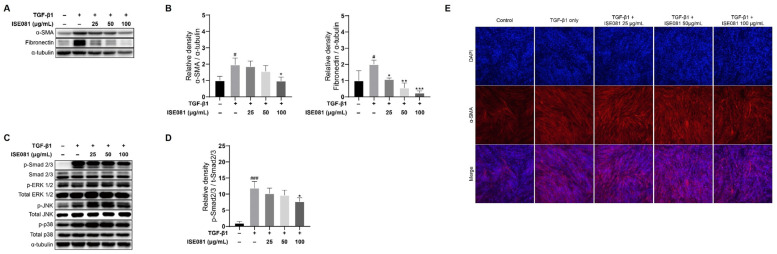
Effect of ISE081 on EMT markers, TGF-β1/Smad signaling, and activation of ERK/JNK/p38 MAPK pathways in MRC-5 cells stimulated with TGF-β1. MRC-5 cells underwent pre-treatment with ISE081 at concentrations of 25 μg/mL, 50 μg/mL, and 100 μg/mL. This was followed by incubation for 24 hours with TGF-β1 at a concentration of 5 ng/mL. The protein levels of α-SMA and fibronectin in cell lysates were determined through Western blot analysis, subsequently quantified and normalized with that of α-tubulin via densitometry analysis (**A**,**B**). Additionally, cells pretreated with ISE081 and then exposed to 5 ng/mL TGF-β1 for 1 h were analyzed for migration (TGF-β1 and Smad2/3) and proliferation (ERK/JNK/p38) signaling proteins using Western blotting and densitometry (**C**,**D**). The phosphorylated forms of Smad2/3 and ERK/JNK/p38 were also normalized in relation to α-tubulin to provide context for each protein expression level. Immunofluorescence staining was employed for α-SMA visualization (**E**), depicting α-SMA in red and cell nuclei in blue with DAPI staining (magnification: 100×). These experiments were conducted in triplicate. The data are shown as mean ± SD with *n* = 3 per group. Statistical significance is indicated as # *p* < 0.05 and ### *p* < 0.001 compared to the control group, and * *p* < 0.05, ** *p* < 0.01, *** *p* < 0.001 compared to the TGF-β1 only group.

EMT is a key factor in the development of PF. This transition is characterized by a decrease in epithelial markers and an increase in mesenchymal markers. In this study, we investigated the impact of ISE081 on EMT marker proteins such as α-SMA and fibronectin in MRC-5 cells. Figure 3A,B demonstrate that treatment with TGF-β1 markedly elevated the levels of α-SMA and fibronectin, while co-treatment with ISE081 effectively reversed these changes. Additionally, immunofluorescence analysis provided supporting evidence for α-SMA expression alterations, as depicted in Figure 3E. Collectively, these findings indicate that ISE081 mitigates the profibrotic effects induced by TGF-β1, potentially through the inhibition of EMT processes.

### 3.4. ISE081 Reduces TGF-β1-Induced Smad2/3 Phosphorylation in MRC-5 Cells

To delineate the impact of ISE081 on TGF-β1-mediated signaling pathways, specifically the Smad2/3 activation and MAPK pathways, we conducted Western blot analyses. These analyses were focused on assessing the phosphorylation levels of key proteins in these pathways, namely Smad2/3, ERK, JNK, and p38. The data presented in Figure 3C,D indicate a significant upregulation of phosphorylated Smad 2/3 in MRC-5 cells subjected to TGF-β1, as compared to the control group. Interestingly, the application of ISE081 was observed to counteract the TGF-β1-induced increase in phosphorylated Smad 2/3 levels. In terms of MAPK signaling involving ERK, JNK, and p38, no inhibitory effect was observed upon ISE081 treatment.

### 3.5. Effect of ISE081 on Cell Migration of Fibroblasts

**Figure 4:**
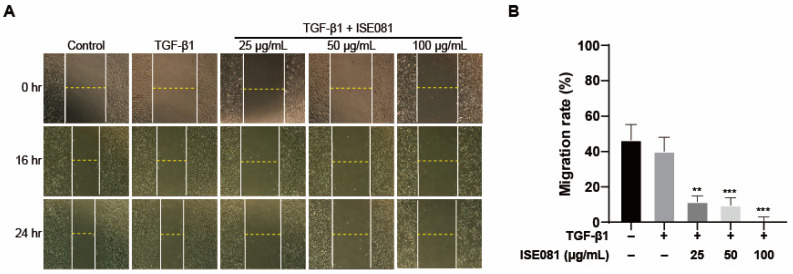
Investigating the influence of ISE081 on MRC-5 cell migration. MRC-5 cells underwent pre-treatment with ISE081 before a 24 h incubation with 5 ng/mL TGF-β1. In comparison, control cells were maintained in standard culture media. The figure presents a representative set of images showing the wound-healing assay results at 0, 16, and 24 h post-scratching (at a magnification of 40×) (**A**). The migration rate was quantified by yielding an invasion index (**B**). The displayed data represent the mean ± SD for three independent experiments in each group. Statistical significance is denoted by ** *p* < 0.01 and *** *p* < 0.001 when compared to the TGF-β1 treated group.

We investigated the influence of ISE081 on the migratory behavior of MRC-5 cells at 0, 16, and 24 h following incubation with both ISE081 and TGF-β1. As depicted in Figure 4A, initially, the control, TGF-β1-treated, and ISE081-treated wells displayed comparable areas of cell clearance. However, after a 24 h period, the areas exhibiting cell absence showed a more pronounced decrease in the wells that received ISE081 treatment at all tested concentrations, relative to the control group (Figure 4A). Notably, the rate of cell migration exhibited a dose-dependent reduction in response to varying concentrations of ISE081, as shown in Figure 4B.

### 3.6. Synergistic Effect of ISE081 Following P. ginseng and I. japonica Formula

**Figure 5:**
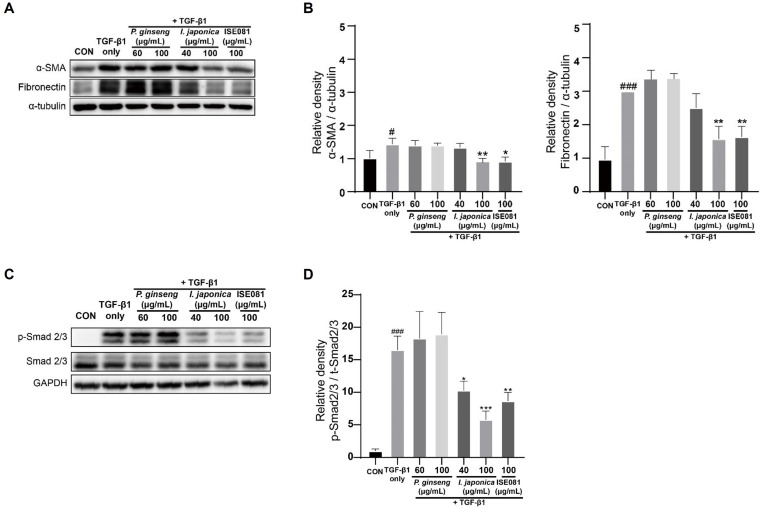
Evaluation of the anti-fibrotic effects of ISE081 relative to each *P. ginseng* and *I. japonica*. MRC-5 cells received pre-treatment with 100 μg/mL ISE081, 60 μg/mL *P. ginseng*, and 40 μg/mL *I. japonica*, respectively, followed by a 24 h incubation with 5 ng/mL TGF-β1. The protein levels of α-SMA and fibronectin in cell lysates were determined through Western blot analysis, subsequently quantified and normalized with that of α-tubulin via densitometry analysis (**A**,**B**). Additionally, cells pretreated with ISE081 and then exposed to 5 ng/mL TGF-β1 for 1 h were subjected to Western blot analysis for Smad2/3. The phosphorylated forms of Smad2/3 were quantified and normalized with that of GAPDH via densitometric analysis (**C**,**D**). The data represent mean ± SD for three replicates in each group. # *p* < 0.05 and ### *p* < 0.001 vs. control group, * *p* < 0.05, ** *p* < 0.01, *** *p* < 0.001 vs. TGF-β1 group.

ISE081 (100 μg/mL) contained *P. ginseng* (60 μg/mL) and *I. japonica* (40 μg/mL) (1.5:1 ratio). The effects of *P. ginseng* and *I. japonica*, which are components of ISE081, on EMT markers were individually confirmed (Figure 5A,B). *P. ginseng* (60 μg/mL) and *I. japonica* (40 μg/mL) did not differ significantly in α-SMA and fibronectin levels compared to the TGF-β1 group. *P. ginseng* (100 μg/mL) treatment showed no significant difference, but *I. japonica* (100 μg/mL) treatment significantly reduced α-SMA and fibronectin levels, and these results were similar to the ISE081 (100 μg/mL) treatment. However, *I. japonica* showed cytotoxicity at 100 μg/mL (Figure 1B). These findings suggest that combining *P. ginseng* and *I. japonica* can have a synergistic effect that inhibits EMT. Additionally, the effects of *P. ginseng* and *I. japonica*, which are components of ISE081, on TGF-β1/Smad signaling were individually confirmed (Figure 5C,D). When compared with the TGF-β1 group, *P. ginseng* treatment did not markedly differ in the p-Smad2/3 level. However, *I. japonica* treatment significantly reduced the p-Smad2/3 level, suggesting that ISE081 inhibition of p-Smad 2/3 was caused by *I. japonica*.

### 3.7. HPLC Analysis for P. ginseng and I. japonica Identification in ISE081

**Figure 6:**
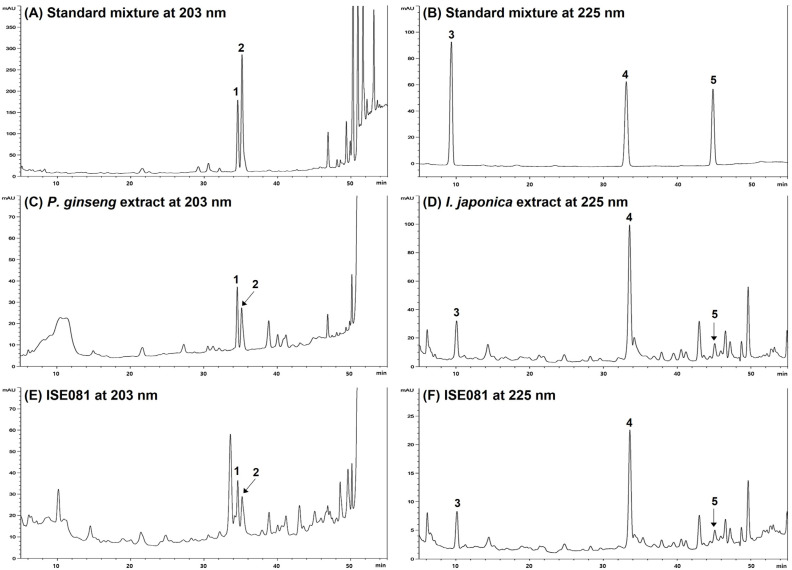
Respective HPLC chromatograms of standard solutions of *P. ginseng* and *I. japonica* (**A**,**B**), 70% ethanol extract of *P. ginseng* and *I. japonica* (**C**,**D**), and ISE081 (**E**,**F**) at 203 nm and 225 nm, respectively. **1**, ginsenoside Rg1; **2**, ginsenoside Re; **3**, Britannilactone; **4**, 1-*O*-Acetylbritannilactone; **5**, 2-α-Hydroxyeudesma-4,11(13)-dien-12,8β-olide.

Under the chromatographic conditions applied in this study, we determined the unique HPLC retention times (*t_R_*) and UV detection wavelengths for the five reference compounds. These parameters were essential for identifying the constituents of the ISE081 formula, which is a blend of *P. ginseng* and *I. japonica.* In Figure 6, the retention times (*t_R_*) of ginsenoside Rg1 (**1**) and Re (**2**) in the standard mixture at 203 nm were 34.57 and 35.15 min, respectively (Figure 6A). And the retention times (*t_R_*) of Britannilactone (**3**), 1-*O*-Acetylbritannilactone (**4**), and 2-α-Hydroxyeudesma-4,11(13)-dien-12,8β-olide (**5**) in the standard mixture at 225 nm were 9.36, 33.09, and 45.09 min, respectively (Figure 6B). Under the same conditions, the components **1** (34.56 min) and **2** (35.16 min) were observed at same retention time in *P. ginseng* extract (Figure 6C), and the components **3** (10.09 min), **4** (33.54 min), and **5** (45.09 min) were observed in *I. japonica* extract (Figure 6D). Based the previous data, the five components were identified in ISE081 by comparing their retention times and UV absorbances with those of the standard compounds (Figure 6E,F).

## 4. Discussion

Notably, various cytokines involved in inflammatory and immune responses play a role in lung fibrosis formation and development [20]. TGF-β1 is a key factor that promotes lung fibrosis and regulates the assembly and remodeling of ECM and EMT [21]. TGF-β is considered a major profibrogenic cytokine, and IL-8 is known to act primarily as a proinflammatory agent, whereas IL-6 contributes to both inflammation and fibrosis [22]. PF acute exacerbations are characterized by abnormally high levels of IL-6 and IL-8. Furthermore, an increase in these cytokines exacerbates patient symptoms and is associated with death [23]. Proinflammatory cytokines, such as IL-6, are involved in fibronectin production [24]. IL-6 production by fibroblasts is induced by TGF-β1 in vitro [25]. This indicates that ECM synthesis in fibrosis activates the proinflammatory pathway. *P. ginseng* and its compound, ginsenosides, have demonstrated anti-inflammatory effects in various in vivo and in vitro studies. Ginsenosides are glucocorticoid receptor (GR) ligands that bind to TLR-4. Partial GR agonists inhibit inflammation without serious side effects [26]. *I. japonica*’s anti-inflammatory effects have been reported in several studies. Notably, britanin isolated from Inulae flos, a flower of *I. japonica*, inhibits the expression of proinflammatory cytokines by suppressing NF-κB and MAPK kinase activity [27,28]. Zhao et al. reported that *I. japonica* extract inhibited the expression level of cytokines, such as TGF-β1 and IL-6, in bleomycin-induced mice [15]. Our findings showed that ISE081 significantly lowered the mRNA expression of cytokines related to inflammation, such as IL-6, IL-8, and TGF-β1 (Figure 1). These anti-inflammatory effects are expected to originate from two sources, *P. ginseng* and *I. japonica*, and are thought to inhibit lung fibrosis formation and development due to their anti-inflammatory effects.

EMT is essential in PF [29]. When various fibrosis factors are activated, the contraction and migration of lung fibroblasts are promoted. Then, a large amount of ECM, such as α-SMA and fibronectin, is deposited [30]. In vitro, exposure to TGF-β1 causes diverse lung epithelial cells to acquire a mesenchymal phenotype [31,32,33]. *P. ginseng* extract and herbal preparations using *P. ginseng* as the dominant drugs inhibit IPF [18]. Furthermore, α-SMA is reduced by ginsenoside Rg1, one of the main active ingredients of *P. ginseng* [34]. Heat-processed *P. ginseng* and ginsenoside Rg3 can downregulate fibronectin expression [35]. However, in our study, when only *P. ginseng* was treated, the expression levels of the EMT markers, such as α-SMA and fibronectin, were not inhibited (Figure 5). *I. japonica*, which has been used for a long time to relieve cough and dyspnea, can exhibit antifibrotic effects by inhibiting the activity of soluble epoxide hydrolase that regulates the glycogen synthase kinase 3β signaling pathway [15]. While *I. japonica* extract inhibited the α-SMA level in BLM-induced mice [15], it also suppressed α-SMA and fibronectin expression in TGF-β1-induced lung fibroblasts [16], which corroborated our findings that showed that α-SMA and fibronectin expression was lowered when *I. japonica* 100 μg/mL was treated in TGF-β1-induced MRC-5 cells (Figure 5A,B). However, 100 μg/mL of *I. japonica* showed cytotoxicity (Figure 1B). ISE081 treatment, in which *P. ginseng* and *I. japonica* were bound in a 1.5:1 ratio, did not show cytotoxicity at a concentration of 100 μg/mL (Figure 1B) and significantly inhibited fibronectin and α-SMA levels in TGF-β1-induced MRC-5 cells (Figure 5A,B). These results suggest that when *P. ginseng* and *I. japonica* were used together, the synergistic effect reduced toxicity and improved antifibrotic activity.

Recent studies have indicated that the overactivation of the TGF-β1/Smad signaling pathway plays a critical role in the development of pulmonary fibrosis [36]. TGF-β1 initiates its signaling process by binding to the TGF-β type II receptor (TβRII), which in turn phosphorylates the TGF-β type I receptor (TβFI). This phosphorylation leads to the formation of heterotetrameric complexes involving TβFI and TβRII, resulting in the phosphorylation of Smad2/3. Beyond the Smad pathway, TGF-β1 is also known to trigger other signaling routes like MEK/ERK, Wnt/β-catenin, and PI3K/Akt [37,38]. Research by Ahn et al. revealed that extracts from P. ginseng could suppress TGF-β1-induced fibrosis by inhibiting the phosphorylation of Smad2 and Smad3 [39]. Furthermore, studies have shown that ginsenosides can reduce EMT by targeting the TGF-β1/Smad pathway [34]. Extracts from I. flos and I. japonica have been found to obstruct the TGF-β1/Smad3 signaling pathway [16,40]. While ISE081 demonstrated a significant suppression of the TGF-β1/Smad signaling pathway, it did not exhibit notable effects on the MAPK pathway, as shown in Figure 3. The evidence suggests that ISE081 may reduce EMT by acting on the TGF-β1/Smad signaling pathway.

Our findings revealed that when *P. ginseng* and *I. japonica* were combined, they exhibited a greater antifibrotic impact than when administered separately. According to TM theory, the main symptoms of patients with pulmonary fibrosis are hematoma, sputum, and lung-qi deficiency. Many oriental doctors believe that these symptoms could be improved by invigorating the lungs, clearing phlegm, and relieving stasis [40]. *P. ginseng* is a widely known herbal medicine that maintains body homeostasis and increases vitality [41]. *I. japonica* has long been used to relieve cough and shortness of breath [42,43]. When used alone, *P. ginseng* has an insignificant antifibrotic effect, but when combined with *I. japonica*, it is thought to have a synergistic effect that lowers toxicity and replenishes the host’s energy, thereby enhancing the antifibrotic ability of *I. japonica*.

## 5. Conclusions

Taken together, our results suggest that combining *P. ginseng* and *I. japonica* enhances the antifibrotic activity in pulmonary fibroblasts while reducing the toxicity typically associated with using a single herbal medicine. This combination appears to exert a synergistic effect against pulmonary fibrosis. Therefore, ISE081 has the potential as a candidate for the prevention and treatment herbal medicine for PF. However, additional studies are required, such as confirming the synergistic antifibrotic effect of ISE081 in a PF animal model through the combination of two herbal medicines for clinical application.

## Data Availability

Data are contained within the article.
